# Oral administration of *D*-glucosamine confers broad-spectrum protection against human coronaviruses including SARS-CoV-2

**DOI:** 10.1038/s41392-023-01483-8

**Published:** 2023-06-13

**Authors:** Qi Qi, Qi Chen, Yumei Dong, Kun Wang, Jialu Wang, Guiming Jin, Aiping Zheng, Rong Zhang, Yongqiang Deng, Yuhuan Li, Chengfeng Qin, Xiaotao Duan

**Affiliations:** 1 State Key Laboratory of Toxicology and Medical Countermeasures, Beijing Institute of Pharmacology and Toxicology, Beijing, China; 2 State Key Laboratory of Pathogen and Biosecurity, Beijing Institute of Microbiology and Epidemiology, Beijing, China; 3 General Hospital of PLA Central Theater Command Department of Disease Prevention and Control, Wuhan, China; 4 Institute of Medicinal Biotechnology, Chinese Academy of Medical Science and Peking Union Medical College, Beijing, China; 5 School of Basic Medical Sciences, Fudan University, Shanghai, China

**Keywords:** Infection, Innate immunity

**Dear Editor**,

The novel human coronavirus (HCoV) SARS-CoV-2 is the causative agent of the current pandemic COVID-19, posing a huge threat to global public health.^1^ Up to now, seven HCoVs have been identified. These HCoVs (including SARS-CoV, HCoV-229E, HCoV-NL63, HCoV-OC43, HCoV-HKU1, MERS-CoV, and SARS-CoV-2) cause a range of symptoms from the common cold to severe pathologies.^2^ As new HCoVs continuously emerge in the human population, there is an urgent need for developing broad-spectrum antiviral therapies that could be effective against HCoVs including SARS-CoV-2 and its emerging variants.

SARS-CoV-2 belongs to positive-sense, single-stranded, enveloped RNA virus, and induces interferons (IFNs) response in host cells.^3^ This antiviral response is primary mediated by retinoic acid-inducible gene I (RIG-I) like receptors (RLRs) -mitochondrial antiviral signaling protein (MAVS) signaling. Once activated, MAVS aggregates and recruits TANK-binding kinase 1 (TBK1), which phosphorylates interferon regulatory factor 3 (IRF3). Phosphorylated IRF3 translocates into the nucleus to activate IFN expression.^4^ IFNs drive the expression of IFN-stimulated genes (ISGs) to control SARS-CoV-2 infection. Clinical evidence has suggested that defects in responsiveness to type I interferon (IFN-I) is of prime importance in determining the severity of SARS-CoV-2 patients,^5^ underlining the significance of IFN signaling in controlling SARS-CoV-2 infection.

We recently identified that *O*-GlcNAcylation, a posttranslational modification derived from hexosamine biosynthetic pathway (HBP), is essential for virus-induced MAVS activation and IFN signaling. We demonstrated that *D*-glucosamine (GlcN), a commonly used dietary supplement, increases MAVS *O*-GlcNAcylation and enhances MAVS-mediated IFN signaling, and thereby exhibits a broad-spectrum antiviral activity.^6^ In this communication, we explore the potential broad-spectrum antiviral activity of GlcN against HCoVs. We first established SARS-CoV-2 infection model using human lung epithelial cell line Calu-3 and human liver cancer cell line Huh7, respectively. Calu-3 or Huh7 cells were treated with GlcN (at a final concentration of 20 mM) for 3 h, infected with SARS-CoV-2 at an multiplicity of infection (MOI) of 1. Upon infection for 24 h, we observed that SARS-CoV-2 infected cells showed a significantly enhanced intensity of cellular *O*-GlcNAcylation comparing to the non-infected group (Fig. 1a, Supplementary Fig. 1), indicating SARS-CoV-2 promotes HBP metabolism and protein *O*-GlcNAcylation in host cells. This is akin to our previous observation in RNA virus infection including IAV and VSV.^6^ As expected, we found that GlcN significantly increased the cellular level of *O*-GlcNAcylation and substantially suppressed SARS-CoV-2 replication in infected lung epithelial cells as measured by SARS-CoV-2 spike protein expression (Fig. 1a). Virus titers in the supernatant were significantly reduced (P < 0.01) in GlcN treatment group (Fig. 1b). Time course measurements (6 to 24 h post infection) further confirmed our observation, GlcN treatment significantly inhibited replication of SARS-CoV-2, consistent with titer reduction (Fig. 1c, d).

**Fig. 1:**
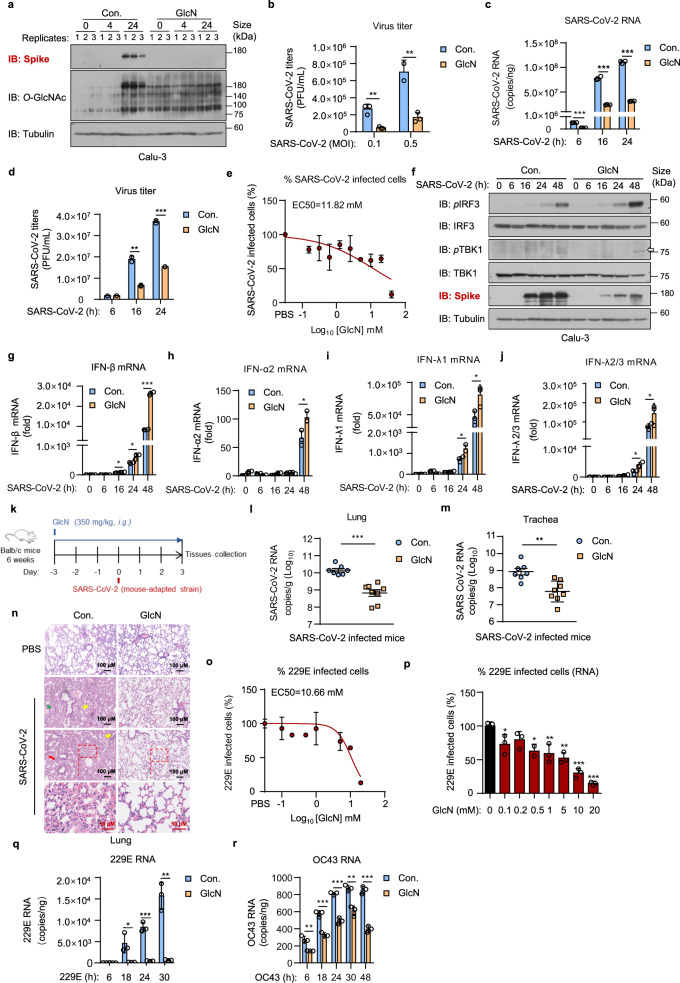
GlcN enhances SARS-CoV-2 induced IFNs signaling and exhibits a broad-spectrum antiviral activity against multiple HCoVs. **a** Calu-3 cells were treated with GlcN for 3 h at 20 mM, infected with SARS-CoV-2 (MOI = 1) for 4 and 24 h. Immunoblotting of SARS-CoV-2 spike and *O*-GlcNAcylation level were performed. **b** Calu-3 cells were treated with GlcN for 3 h at 20 mM, infected with SARS-CoV-2 at an MOI of 0.1 or 0.5. Infectious supernatant was collected at 24 h post infection. Virus titers were measured. **c**, **d** Calu-3 cells were treated with GlcN for 3 h at 20 mM, infected with SARS-CoV-2 (MOI = 1) for 6, 16 and 24 h. Analysis of SARS CoV-2 RNA level in cells **c**, virus titer in supernatant **d** were performed. **e** Calu-3 cells were treated with GlcN ranging from 0.1 to 40 mM for 3 h, infected with SARS-CoV-2 (MOI = 1) for 24 h. Cells were stained for the SARS-CoV-2 N protein and imaged. The percentage of SARS-CoV-2 staining was quantified as shown. **f**–**j** Calu-3 cells were treated with GlcN for 3 h at 20 mM, infected with SARS-CoV-2 for 6, 16, 24, and 48 h. Immunoblotting of SARS-CoV-2 spike, phosphorylated IRF3 and TBK1 were performed **f**. qRT-PCR analysis of IFN-β **g**, IFN-α2 **h**, IFN-λ1 **i**, and IFN-λ2/3 **j** expression was performed. The virus titer of SARS-CoV-2 used in (**a**, **c, d, f**–**j**) was 3.5 × 10^6^ PFU/mL, used in **b**, **e** was 1.73 × 10^6^ PFU/mL. **k** Schematic diagram of the mice experiment. 6 weeks old Balb/c mice were intragastric administration of GlcN (350 mg/kg) for 3 days. Mice were infected with SARS-CoV-2 strain MASCp6 intranasally. Infection for 3 days, mice were sacrificed and tissues were collected. **l**, **m** mice were treated as **k**. qRT-PCR analysis of SARS-CoV-2 RNA level in lung **l** and trachea **m** sections were performed. **n** H&E-stained analysis showing inflammatory cell infiltration (red arrow), alveolar septal thickening (yellow arrow) and fibrin exudation (green arrow) in lungs from mice treated as **k**. The virus titer of SARS-CoV-2 used in **l**–**n** was 4 × 10^5^ PFU/mL. **o, p** Calu-3 cells were treated with GlcN ranging from 0.1 to 20 mM for 3 h, infected with 229E (1 × 10^7^ PFU/mL) at an MOI of 1 for 24 h. RNA and infectious supernatant were collected. Dose-response curves of GlcN is shown and EC50 is indicated above the curve **o**. The percentage of 229E infection calculated by viral RNA is shown as indicated **p**. **q**, **r** Calu-3 cells were treated with GlcN for 3 h at 20 mM, infected with 229E or OC43 at an MOI of 1 for 6, 18, 24, 30 h. qRT-PCR analysis of 229E **q** and OC43 **r** RNA expression was performed. qRT-PCR results are presented relative to those of GAPDH **g**–**j**. Data were shown as means ± SEM **l**, **m**, means ± SD **b**–**e**, **g**–**j, o**–**r** from triplicates (biological replicates), *p < 0.05, **p < 0.01, and ***p < 0.001 (two-tailed Student’s t test)

We next determined the median effective concentration (EC50) value of GlcN against SARS-CoV-2 infection. Calu-3 cells were treated with various doses of GlcN ranging from 0.1 to 40 mM for 3 h, infected with SARS-CoV-2 at an MOI of 1. Infection for 24 h, cells were fixed, stained for the SARS-CoV-2 nucleocapsid (N) protein. After staining, the cells were imaged (Supplementary Fig. 1b). The fraction of infected cells was quantified. GlcN inhibited SARS-CoV-2 infection in Calu-3 cells with a EC50 value of 11.82 mM (Fig. 1e). The cytotoxicity of GlcN was examined in parallel with antiviral activity. We did not observe any measurable cytotoxicity at all concentrations (Supplementary Fig. 1c). These results suggest that GlcN effectively inhibits SARS-CoV-2 replication and infection in vitro.

In an effort to better understand the antiviral mechanism of GlcN against SARS-CoV-2, we examined the SARS-CoV-2 induced interferon response in the presence of GlcN. We observed that GlcN treatment resulted in a considerable increase of both phosphorylated IRF3 and phosphorylated TBK1, and thereby promoted IFN signaling in response to SARS-CoV-2. This in turn decreased the expression of SARS-CoV-2 spike protein in GlcN treatment group (Fig. 1f). It is worth to note that in addition to type I IFN (*i.e*. IFN-β, IFN-α2), we also observed a significant elevation of type III IFN (IFN-λ1, IFN-λ2/3) in GlcN treatment group upon SARS-CoV-2 infection (Fig. 1g–j). This is in line with our previous finding that GlcN promotes MAVS-mediated IFN production.^6^ While mitochondrial MAVS preferentially mediates the expression of type I IFNs, peroxisomal MAVS selectively activates IFN-λ.^7^ This provides a better rationale for GlcN treatment, as SARS-CoV-2 is sensitive to both type I and type III IFNs.^8^ Consequently, we found that IFITM1, IFIT2/ISG54 and CXCL10 were significantly elevated in GlcN treatment group comparing to control (Supplementary Fig. 2a–c), further confirming the proposed mechanism. These findings together provide sufficient evidence that GlcN promotes host antiviral IFN pathway against SARS-CoV-2.

To evaluate the antiviral effect of GlcN against SARS-CoV-2 in vivo, we first assessed the safety of GlcN. Mice were intragastric administrated with GlcN (350 mg/kg) for 10 consecutive days. Body weights were measured daily, and we did not detect any body weight loss of mice in GlcN-treatment group (Supplementary Fig. 3a). We also collected the lungs, spleens and livers from mice, and the orally administrated GlcN did not induce systemic IFN response in vivo (Supplementary Fig. 3b–d). H&E staining showed that GlcN did not cause any pathological changes in intestinal tract (Supplementary Fig. 3e). On the basis of these safety data, we established a mouse-adapted SARS-CoV-2 infected model.^9^ 6 weeks old BALB/c mice were intragastric administrated with GlcN (350 mg/kg) for 3 consecutive days, and were then intranasally inoculated with SARS-CoV-2 strain MASCp6. On day 3 post-infection, mice were sacrificed to assess viral loads and inflammation in trachea and lung (Fig. 1k). GlcN treatment resulted in a greater than 20-fold reduction in SARS-CoV-2 RNA in the lungs and a 10-fold reduction in the tracheas (Fig. 1l, m). Pulmonary histologic examination by H&E staining showed that SARS-CoV-2-infected mice developed interstitial pneumonia characterized with inflammatory cell infiltration, alveolar septal thickening, and peribronchiolar inflammation. Compared to the control group, we observed that GlcN treatment profoundly reduced the inflammatory infiltrate and epithelial damage caused by SARS-CoV-2 infection (Fig. 1n). These results showed that orally administrated GlcN restricts the replication of SARS-CoV-2 and alleviates virus-induced lung injury in vivo.

To assess the broad-spectrum activity of GlcN against HCoVs, we established cellular infection models using HCoV-229E (229E) and HCoV-OC43 (OC43), respectively. We first determined the EC50 values of GlcN against 229E infection. GlcN inhibited 229E infection in Calu-3 cells with a EC50 value of 10.66 mM (Fig. 1o). We also assessed the abundance of intracellular viral RNA via qRT-PCR. A reduction of 229E RNA was observed upon GlcN treatment (Fig. 1p). We next explored the antiviral activity of GlcN against HCoVs over the time course of 229E/OC43 infection. We found that GlcN treatment resulted in a pronounced reduction (~ 95%) of 229E RNA level at 18, 24 and 30 h post-infection (Fig. 1q). Likewise, GlcN treatment greatly inhibited OC43 RNA replication in Calu-3 cells. (Fig. 1r, Supplementary Fig. 4a). Ribavirin was used as a positive control in both assessments. GlcN showed comparable efficacy versus ribavirin in terms of HCoVs replication (Supplementary Fig. 4b, c), suggesting a broad-spectrum antiviral activity against diverse HCoVs.

Taken together, this study demonstrated that GlcN enhances SARS-CoV-2 induced IFNs signaling and restricts SARS-CoV-2 replication in multiple human cell lines. In SARS-CoV-2 infected mouse model, prophylactic administration of GlcN at a clinical-relevant dose significantly reduces the viral load in lung and trachea, and considerably alleviates lung inflammation. A recent clinical study (*ClinicalTrials.gov*: NCT04706416) reported that orally administrated *N*-acetyl glucosamine (NAG), a downstream metabolite of GlcN, decreases the mortality rate and improves clinical outcomes of SARS-CoV-2 infected patients.^10^ Despite a larger-scale multi-center trial is still underway, the pilot data are generally in line with our experimental results. It is also important to note that GlcN exhibited broad-spectrum anti-viral activity against HCoVs, including 229E and OC43. The antiviral effect of GlcN is stronger against 229E infection than OC43. Apparently, 229E and OC43 induce host interferon response to a different extent. 229E infection induced significantly higher level of type I and type III IFNs compared with OC43.^11^ We therefore reasoned that the relative mild antiviral effect of GlcN against OC43 is due to the less extent of interferon-mediated innate immune response that OC43 induced in host cells.

Overall, our work demonstrated that GlcN shows potential efficacy against multiple HCoVs including SARS-CoV-2, 229E and OC43 in both cell-based and mouse infection model. GlcN has been clinically applied for the treatment of osteoarthritis for more than 50 years. As a nutrient supplement, orally administrated GlcN at daily-doses ranging from 750 to 3500 mg is well tolerated in human subjects. Given the safety profile and its broad-spectrum anti-HCoVs activity, GlcN may serve as a promising drug for preventing the spread of SARS-CoV-2 and its emerging variants in healthy populations.

## Supplementary information

Supplementary Information for Oral administration of D-glucosamine confers broad-spectrum protection against human coronaviruses including SARS-CoV-2

## Data Availability

All data are available from the corresponding author upon reasonable request.
