# Supplementary material for: Oral administration of D-glucosamine confers broad-spectrum protection against human coronaviruses including SARS-CoV-2
Source: Signal Transduct Target Ther. 2023 Jun 13;8:250. doi: 10.1038/s41392-023-01483-8 (PMC10262929; doi:10.1038/s41392-023-01483-8)
Supplement: Supplementary file 1 — Supplementary Information for Oral administration of D-glucosamine confers broad-spectrum protection against human coronaviruses including SARS-CoV-2 [file 41392_2023_1483_MOESM1_ESM.docx]

Supplementary Information for

Oral administration of *D*-glucosamine confers a broad-spectrum protection against human coronaviruses including SARS-CoV-2

Qi Qi^1^, Qi Chen^2^, Yumei Dong^3^, Kun Wang^4^, Jialu Wang^3^, Guiming Jin^3^, Aiping Zheng^1^, Rong Zhang^5^, Yongqiang Deng^2^, Yuhuan Li^4^, Chengfeng Qin^*2^ and Xiaotao Duan^*1^

^1^ State Key Laboratory of Toxicology and Medical Countermeasures, Beijing Institute of Pharmacology and Toxicology, Beijing 100850, China

^2^ State Key Laboratory of Pathogen and Biosecurity, Beijing Institute of Microbiology and Epidemiology, Beijing 100850, China

^3^ General Hospital of PLA Central Theater Command Department of Disease Prevention and Control, Wuhan 430070, China

^4^ Institute of Medicinal Biotechnology, Chinese Academy of Medical Science and Peking Union Medical College, Beijing 100050, China

^5^ School of Basic Medical Sciences, Fudan University, Shanghai 200032, China

These authors contributed equally: Qi Qi, Qi Chen, Yumei Dong

^*^Correspondence: Xiaotao Duan ([xduan@ncba.ac.cn](mailto:xduan@ncba.ac.cn)); Chengfeng Qin ([qingcf@bmi.ac.cn](mailto:qingcf@bmi.ac.cn))

**This PDF file includes:**

Materials and Methods

Supplementary Figures S1 to S4, and legends

Supplementary Table

**Materials and Methods**

**Cells**

Calu-3 cells (Procell, CL-0054) was cultured in Minimum Essential Medium (MEM) supplemented with 10% FBS, 1% penicillin-streptomycin. Huh7 cells were kindly provided by Dr. Ruiyuan Cao and were cultured in Dulbecco's Modified Eagle Medium (DMEM) supplemented with 10% FBS, 1% penicillin-streptomycin. Cells were cultured at 37 °C with 5% CO_2_.

**Viruses propagation and titeration**

SARS-CoV-2 strain (patient-derived isolates) was originally isolated by Chengfeng Qin’s lab. The virus was amplified on Vero cells and titerated by standard plaque forming assay. Briefly, Vero cells in 12-well plates were infected with a 10-fold serial dilution of viruses. The plates were incubated at 37 °C for 1 hour and cells were overlaid with 1% low-melting point agarose (Promega) in DMEM containing 2% FBS. After further incubation at 37 °C for 2 days, the cells were fixed with 4% formaldehyde and stained with 0.2% crystal violet to visualize the plaques. All experiments involving infectious SARS-CoV-2 were performed in biosafety level 3 (BSL3) facility. HCoV-229E and HCoV-OC43 were kindly provided by Dr. Yuhuan Li (Institute of Medicinal Biotechnology, Chinese Academy of Medical Sciences and Peking Union Medical College). The experiments with HCoV-229E and HCoV-OC43 were performed at BSL-2 facility.

**RNA isolation and qRT-PCR assays**

Total RNA was extracted by TRIzol Reagent (Sigma, T9424). RNA concentration was measured by NanoDrop Spectrophotometer (Thermo Scientific). 500 ng total RNA was reversed-transcribed using the Reverse Transcription System from TaKaRa (RR036A-1). qRT-PCR assays were conducted with SYBR Green Master Mix (Thermo Fisher, A25742). To detect IFN signaling activation, the ddCt method was used to calculate the relative change of gene expressions. Quantitation of target gene was normalized to GAPDH. To detect virus RNA expression, synthetic viral fragments were used to make a standard curve. The primer sequences for qRT-PCR analysis were synthesized from Sangon Biotech and listed in Supplementary Table.

**Antibodies**

The antibodies used in this research were: Anti-phosphorylated IRF3 (*p*IRF3) from Abcam (ab76493); Anti-IRF3 from Abcam (ab68481); Anti-phosphorylated TBK1 (*p*TBK1) from Cell signaling Technology (5483S); Anti-TBK1 from Cell signaling Technology (3504s); Anti-SARS-CoV-2 Spike S2 from Invitrogen (MA5-35946); Anti-*O*-GlcNAcylation from Abcam (ab2739).

**Immunoblotting**

For immunoblotting, cells were washed with ice-cold PBS and lysed with M2 lysis buffer (20 mM Tris-HCl, pH 7.5, 3 mM EDTA, 3 mM EGTA, 250 mM NaCl, 0.5% NP-40) supplemented with PMSF (Bimake, PA116-01) and cocktail protease inhibitor (Bimake, B14001). Lysates were mixed with sample loading buffer and denatured at 95℃ for 15 minutes. Samples were separated by SDS-PAGE, transferred onto PVDF membrane. Immunoblot analysis was performed with relevant antibodies.

**Virus infection *in vitro***

Cells were pretreated with GlcN (MCE, HY-B1125) at indicated concentrations for 3 hours. The culture medium was switched to 5 mM DMEM for 12 hours before GlcN treatment. Cells were infected with SARS-CoV-2 at a multiplicity of infection (MOI) of 0.1, 0.5 or 1. HCoV-229E and HCoV-OC43 were infected at a MOI of 1. Unbound virus was washed away after 1 hour, and cells were then cultured with fresh medium supplemented with 2% FBS. Infection for indicated time, cells were collected. All experiments with SARS-CoV-2 virus were performed within the BSL-3 facility.

**Virus infection *in vivo***

6-weeks wild-type Babl/c mice were purchased from Charles River. Age and sex matched mice were divided into control and GlcN group. Mice in GlcN group were intragastric administrated of GlcN (Sigma-aldrich, G4875) at 350 mg/kg for 3 days. After 3 days, mice were intranasally infected with a mouse-adapted SARS-CoV-2 strain MASCp6. Infection for 3 days, mice were sacrificed, lung and trachea sections were collected. Lung and trachea sections were weighed and homogenized in TRIzol Reagent (Sigma, T9424). After homogenization, the suspensions were centrifuged at 12000 rpm for 15 minutes. The SARS-CoV-2 RNA level in the lysis was measured by qRT-PCR.

All procedures involving infectious virus were conducted in Biosafety Level 3 laboratory and approved by the Animal Experiment Committee of Laboratory Animal Center, Beijing Institute of Microbiology and Epidemiology (approval number: IACUC-DWZX-2020-002).

**Dose-response analysis for SARS-CoV-2 infection**

Calu-3 cells were treated with various doses of GlcN ranging from 0.1 to 40 mM for 3 hours, infected with SARS-CoV-2 at an MOI of 1. Infection for 24 hours, cells were fixed with 4% paraformaldehyde, blocked in 5% BSA + 0.5% Triton X-100 for 1 hour, immunostained overnight at 4 °C with antibody against SARS-CoV-2 nucleocapsid (N) protein. Confocal imaging was performed and images were analysed to determine the SARS-CoV-2 infected cells. Results are indicative of the percentage of SARS-CoV-2 N protein (yellow) staining cells.

**Dose-response analysis for 229E and OC43 infection**

Calu-3 cells were treated with various doses of GlcN ranging from 0.1 to 20 mM for 3 hours, infected with 229E or OC43 at an MOI of 1. Cells were washed with 1× PBS at 24 hours post infection, infectious supernatants and cellular RNA were collected. The viral RNA level was measured by qRT-PCR. Serial dilutions of each infectious supernatants were added to the Huh7 cells plated in 96-well. After incubation for 3 days, cells were fixed with 4% paraformaldehyde, and stained with 0.1% crystal violet to visualize cytopathic effect (CPE). Wells were scored for CPE and the resulting scores were used to calculate the TCID50. The EC50 values were calculated by fitting a sigmoidal curve onto the data of a dose response curve experiment using the Prism (GraphPad) software.

**Cytotoxicity of GlcN in Calu-3 cells**

For CC50, Calu-3 cells were plated at 10,000 cells per well in a 96-well plate and incubated at 37°C. After 24 hours, various doses of GlcN ranging from 0.1 to 40 mM were added in triplicate. Incubated at 37°C for 48 hours, cell viability was measured via CCK8 assay according to the manufacturer’s instructions. Percent viability values were determined by normalization to PBS-only control wells with background luminescent signal subtracted out.

**Lung histology**

Lungs from SARS-CoV-2-infected mice were collected and were fixed in 10% phosphate-buffered formalin. 5 µm tissue sections were prepared from paraffin embedded tissue. Sections were stained with hematoxylin and eosin solution (H&E) and analyzed by NanoZoomer 2.0HJ. H&E staining analysis of lung from control group included perivascular inflammation, bronchial or bronchiolar epithelial degeneration and alveolar inflammation. These changes were alleviated in lungs from GlcN treated mice.

**Statistical analysis**

EC50 was calculated by fitting a sigmoidal curve onto the data of a dose response curve experiment. Statistical analyses were performed with a two-tailed unpaired t test. P values are indicated by asterisks in the figures as followed: *p < 0.05, **p < 0.01 and ***p < 0.001. Data is presented as mean ± SEM or mean ± SD as indicated in the legend. There was no exclusion of data points or mice.

**Supplementary Fig. S1**

**
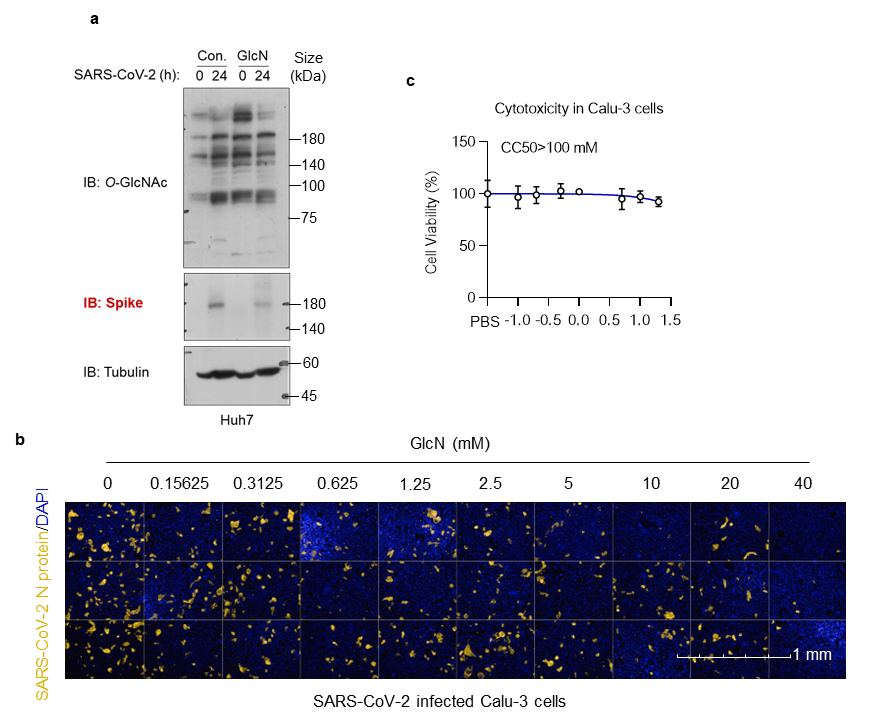
**

**Supplementary Fig. S1 Inhibitory effects of GlcN against SARS-CoV-2. a** Huh7 cells were treated with GlcN for 3 h at 20 mM, infected with SARS-CoV-2 (MOI=1) for 24 h. Immunoblotting of SARS-CoV-2 spike and *O*-GlcNAcylation level were performed. **b** Calu-3 cells were treated with GlcN ranging from 0.1 to 40 mM for 3 h, infected with SARS-CoV-2 (MOI=1) for 24 h. Cells were stained for the SARS-CoV-2 N protein and imaged. Scale bar, 1 mm. **c** Cytotoxicity in Calu-3 cells treated with various doses of GlcN for 48 h. Cell viability was measured via CCK8 assay.

**Supplementary Fig. S2**


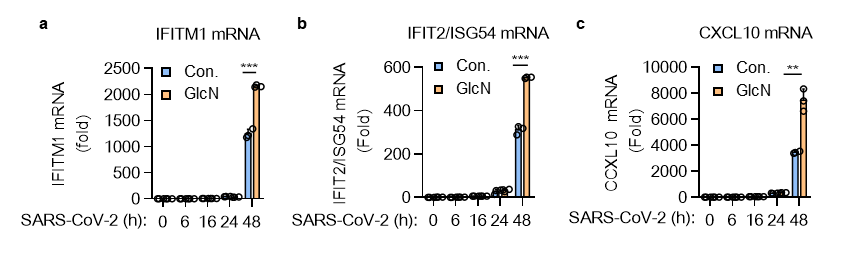


**Supplementary Fig. S2 GlcN enhanced SARS-CoV-2-induced IFN signaling. a-c** Calu-3 cells were treated with GlcN for 3 h at 20 mM, infected with SARS-CoV-2 for indicated time. qRT-PCR analysis of IFITM1 (**a**), IFIT2/ISG54 (**b**) and CXCL10 (**c**) were performed. qRT-PCR results are presented as means ±SD, relative to those of GAPDH.

**
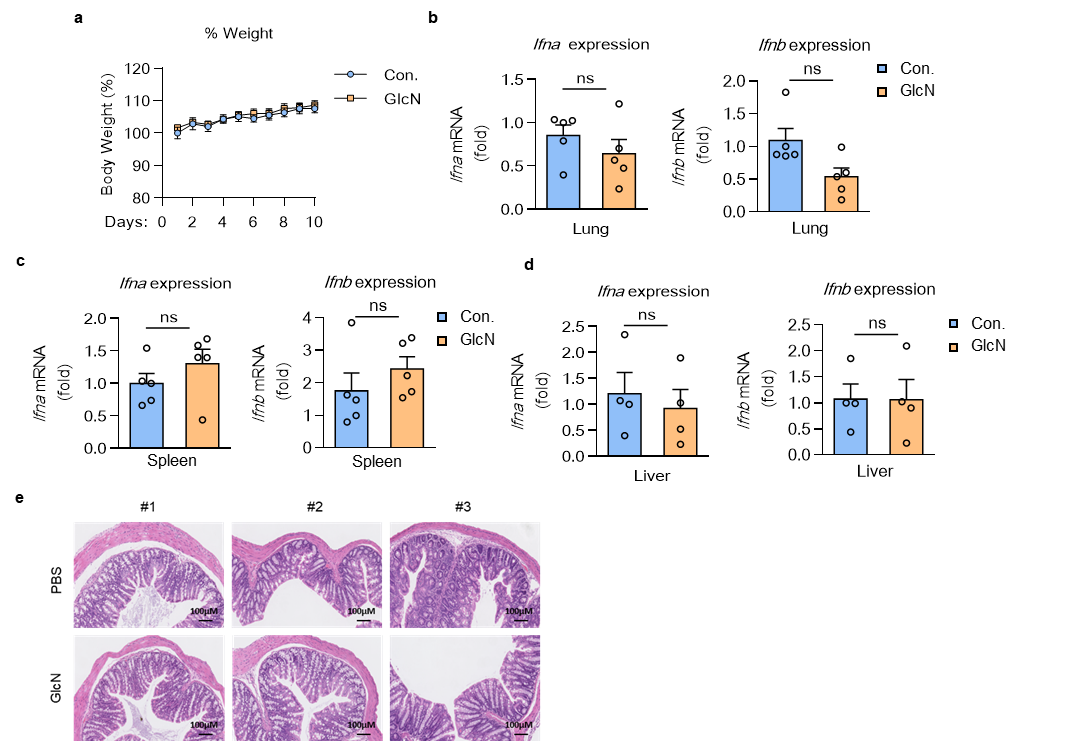
Supplementary Fig. S3**

**Supplementary Fig. S3** **Oral administration of GlcN displayed a safety** **profile** **in mice. a** Wild-type BALB/c mice were intragastric administered of GlcN (350 mg/kg) for 10 consecutive days. Mice in control group were intragastric administered of PBS daily. Percent starting weight of mice is shown as indicated (n=8 per group). **b-d** Mice were treated as (**a**). qRT-PCR analysis of *Ifna* and *Ifnb* RNA in lung (**b**), spleen (**c**) and liver (**d**) (n=5 per group) were performed. **e** Mice were treated as (**a**). H&E-stained analysis of colon was performed (n=3 per group). qRT-PCR results are presented as means ±SD, relative to those of GAPDH (**b-d**).

**
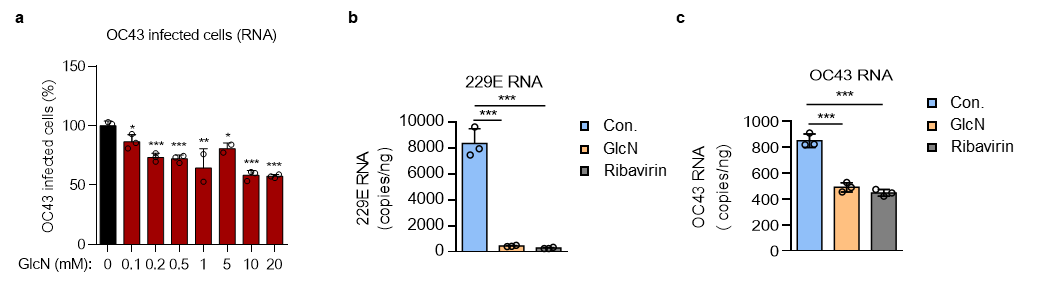
Supplementary Fig. S4**

**Supplementary Fig. S4 Inhibitory effects of GlcN against HCoVs. a**. Calu-3 cells were treated with various doses of GlcN for 3 h, infected with OC43 (MOI=1) for 24 h. The percentage of OC43 infection calculated by viral RNA is shown as indicated. **b, c** Calu-3 cells were treated with GlcN for 3 h at 20 mM, infected with 229E (**b**) or OC43 (**c**) for 24 h. Ribavirin treatment as a positive control. qRT-PCR analysis of viral RNA level was performed. qRT-PCR results are presented as means ±SD.

**Supplementary Table**

| **Gene** | **Forward primer** **(5’-3’)** | **Reverse primer (5’-3’)** |
| --- | --- | --- |
| hIFN-β | CCAACAAGTGTCTCCTCCAAATT | CCAACAAGTGTCTCCTCCAAATT |
| hIFN-α2 | GCTTGGGATGAGACCCTCCTA | GCTTGGGATGAGACCCTCCTA |
| hIFN-λ1 | CACATTGGCAGGTTCAAATCTCT | CCAGCGGACTCCTTTTTGG |
| hIFN-λ2/3 | AGTTCCGGGCCTGTATCCAG | GAGCCGGTACAGCCAATGGT |
| hIFN-λ4 | CGATCCTGGAGCTGCTG | CGATCCTGGAGCTGCTG |
| hIFITM1 | CCAAGGTCCACCGTGATTAAC | CCAAGGTCCACCGTGATTAAC |
| hIFIT2/ISG54 | AAGCACCTCAAAGGGCAAAAC | AAGCACCTCAAAGGGCAAAAC |
| hCXCL10 | TGAAAAAGAAGGGTGAGAAGAGATG | TGAAAAAGAAGGGTGAGAAGAGATG |
| hGAPDH | CCAGGTGGTCTCCTCTGACTTC | CCAGGTGGTCTCCTCTGACTTC |
| m*Ifna* | CCTGAACATCTTCACATCAAAGGA | AGCTGCTGGTGGAGGTCATT |
| m*Ifnb* | CCAAGAAAGGACGAACATTCG | TCCGTCATCTCCATAGGGATCT |
| m*Gapdh* | TGCAGTGGCAAAGTGGAGATT | GTGAGTGGAGTCATACTGGAACATGT |
| SARS-CoV-2 | GGATCAAGAATCCTTTGGTGG | GTCACAAAATCCTTTAGGATTTGGA |
| HCoV-229E | TGAAGATGCTTGTACTGTGGCT | CTGTCATGTTGCTCATGGGG |
| HCoV-OC43 | ATGTCAATACCCCGGCTGAC | GGCTCTACTACGCGATCCTG |
